# Percutaneous Microwave Ablation of Liver Lesions: Differences on the Sphericity Index of the Ablation Zone between Cirrhotic and Healthy Liver Parenchyma

**DOI:** 10.3390/diagnostics11040655

**Published:** 2021-04-05

**Authors:** Athanasios Tsochatzis, Argyro Mazioti, Georgios Iliadis, Georgios Velonakis, Evgenia Efthymiou, Alexis Kelekis, Nikolaos Kelekis, Dimitrios Filippiadis

**Affiliations:** 2nd Department of Radiology, Medical School, University General Hospital “ATTIKON”, National and Kapodistrian University of Athens, 15122 Athens, Greece; thanasis.tsochatzis@hotmail.com (A.T.); argyromazioti@yahoo.gr (A.M.); georgeiliadis@hotmail.co.uk (G.I.); giorvelonakis@gmail.com (G.V.); efthymiouevgenia@gmail.com (E.E.); akelekis@med.uoa.gr (A.K.); kelnik@med.uoa.gr (N.K.)

**Keywords:** microwave ablation, liver, fibrosis, cirrhosis

## Abstract

To compare different parameters of the sphericity index of the ablation zone following microwave ablation (MWA) on cirrhotic- and healthy-liver parenchyma in a series of patients treated with the same MWA system. Institutional database research identified 46 patients (77 lesions) who underwent MWA. “Cirrhotic liver group” (CLG) included 35 hepatocellular carcinoma lesions; “healthy liver group” (HLG) included 42 metastatic lesions. The long axis (LAD), short axis 1 (SAD-1) and 2 (SAD-2), the mean SAD-1 and SAD-2 (mSAD) diameter (in mm) and the mean sphericity (mSPH) index of the ablation zones were evaluated for each treated lesion in both groups from baseline to follow-up. A mixed model analysis of variance reported significant main effect of group on SAD-1 (*p* = 0.023), SAD-2 (*p* = 0.010) and mSAD (*p* = 0.010), with HLG showing lower values compared to CLG. No differences were detected on the LAD (p_FDR_ = 0.089; d = 0.45), and mSPH (p_FDR_ = 0.148, d = 0.40) between the two groups. However, a significant main effect of time was found on LAD (*p* < 0.001), SAD-1 (*p* < 0.001), SAD-2 (*p* < 0.001) and mSAD (*p* < 0.001), with decreased values in all indices at follow-up compared to baseline. A significant group by time interaction was observed on mSPH (*p* = 0.044); HLG had significantly lower mSPH at follow-up where CLG did not show any significant change. Our findings indicate that although in cirrhotic liver short axis diameter of the MWA zone seems to be significantly longer, this has no effect on the sphericity index which showed no significant difference between cirrhotic vs. healthy liver lesions. On the contrary, on one month follow-up ablation zones tend to become significant more ellipsoid in healthy whilst remains stable in cirrhotic liver.

## 1. Introduction

Percutaneous imaging-guided MWA is an increasingly applied technique for the treatment of malignant liver tumors, achieving high local tumor control rates in both primary and secondary lesions irrespective of the target’s histology [1,2,3]. MWA systems have been industrialized and considered as another option to conventional Radiofrequency (RF)-based platforms. Advantages of the MWA systems include the aptitude to reach higher temperatures and greater ablation zones in less time, with less heat sink effect when compared to RF ablation [4,5,6]. When compared to other heat based ablation techniques, microwaves are more effective in tissues with high blood circulation or near vascular structures with resultant ablation zones being relatively unvarying in shape and size and more predictable in extension [7,8].

Presence of hepatic cirrhosis (especially severe one) may affect clinical practice for image-guided percutaneous MWA in liver lesions minimizing the safety margins up to 5 mm [9]. In addition the “oven effect” theory proposed by Liu et al. supports that the increased heating efficacy for tumors surrounded by a cirrhotic liver is attributed to the fibrotic changes that cause the liver to act as a refractive tissue which condensates energy [10]. According to the “oven effect” theory, one might assume that higher amounts of energy deposition would be required in the healthy versus the cirrhotic liver parenchyma in order to create a similar ablation zone. Cassinoto et al. in a recent study clearly proved that in contradiction to “oven effect” theory, the size of the ablation zone following radiofrequency ablation is independent of the presence or absence of cirrhosis [11].

As far as sphericity of the MWA zone is concerned, Hoffmann et al. have concluded that different microwave antennas result in different sphericity indexes. Systems with multiple antennae seem to produce a more spherical ablation zone on the expense of higher session cost and duration as well as of higher potential complication rates due to the additional penetrations of the hepatic capsule that might lead to a higher risk of bleeding [12]. In a more recent study by Cazzato et al., the authors conclude that simultaneous multi-antennae MWA in the liver results in nearly spherical ablation zones [13]. However, the study by Hoffmann et al. was an experimental one in ex vivo bovine liver and there was no comparison between cirrhotic and healthy liver parenchyma [12]. Similarly in the Cazzato et al. study there was no evaluation of any potential effect of the cirrhotic parenchyma upon the sphericity of the ablation zone [13].

The aim of the present study was to compare different parameters of the sphericity index of the ablation zone following MWA on cirrhotic- and healthy-liver parenchyma in a series of patients treated with the same MWA system.

## 2. Materials and Methods

### 2.1. Patient Selection and Evaluation

**Figure 1:**
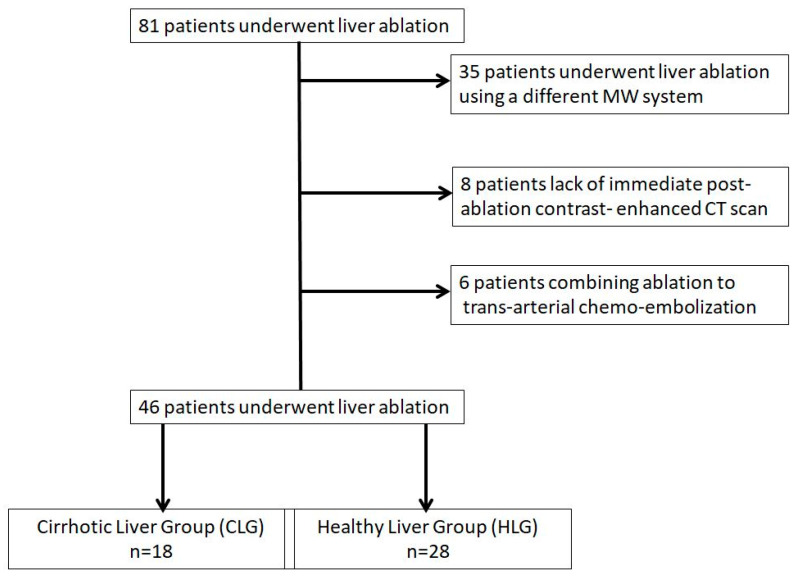
Flow diagram of patient selection for the study.

Institutional database research over a three-year period from 2016 to 2019 identified 81 patients who underwent percutaneous microwave ablation of a hepatic lesion. Inclusion criteria for the present study were: (1) age ≥ 18 years with primary or metastatic liver disease confirmed either by prior biopsy with a histopathological diagnosis or through imaging [a combination of with or without contrast-enhanced ultrasound (US), contrast-enhanced computed tomography (CT) and/or magnetic resonance imaging (MRI)], (2) MWA using the same system (antenna and generator), (3) lack of a combined therapy such as ablation and trans-arterial chemoembolization (conventional or with drug eluting beads), (4) baseline contrast-enhanced CT or MRI scan of less than one month available and (5) immediate and at 1 month post-ablation contrast- enhanced CT scan. Based on the aforementioned inclusion criteria, 46 patients (mean age 66.49 ± 9.90 years) who underwent percutaneous microwave ablation of 77 liver lesions were eligible for the study. The remaining 35 patients were excluded due to ablation session using a different microwave system (21/35), lack of immediate post-ablation contrast- enhanced CT scan (8/35) and combining ablation to trans-arterial chemoembolization (6/35) (Figure 1).

All patients were selected for microwave ablation by a multidisciplinary team of medical, radiation, surgical and interventional oncologists. The patients were fully informed about the procedure, the possible complications and the surgical and medical alternatives available; informed written consent for the ablation was obtained from all patients. Patients were divided into those with hepatocellular cancer lesions developed in cirrhotic liver [cirrhotic liver group (CLG)] and those with hepatic metastases developed on a healthy liver [healthy liver group (HLG)] based on medical record and imaging findings on CT (including signs of cirrhosis and portal hypertension). Each patient underwent laboratory tests (including renal function and coagulation tests) at least 24 h prior to the percutaneous ablation session.

### 2.2. Percutaneous MW Ablation Procedure

**Figure 2:**
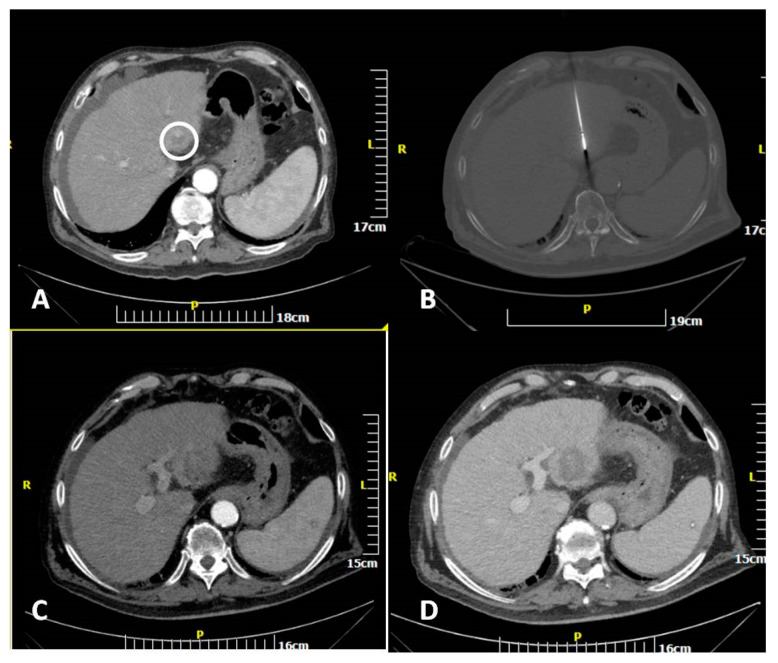
CT scan of an 82-year-old man showing a hepatocellular cancer lesion. Enhanced transverse (**A**) CT image illustrates the lesion (white circle). Transverse CT image (**B**) illustrating the microwave antenna at the lesion level. Transverse CT images in arterial (**C**) and portal venous (**D**) phases illustrating the ablation zone.

**Figure 3:**
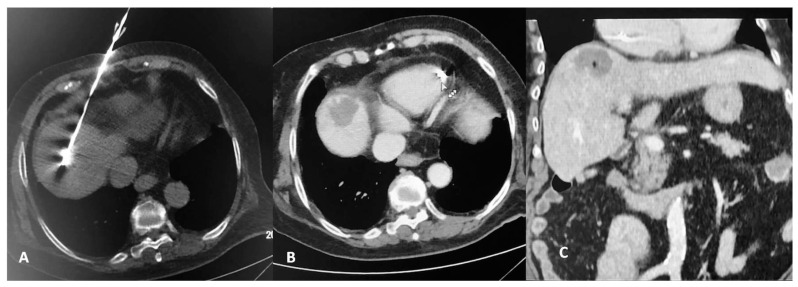
CT scan of an 83-year-old female patient with a solitary colorectal cancel metastatic lesion at hepatic segment VIII. CT axial reconstruction (**A**) illustrating the microwave antenna at the lesion level. Transverse CT image in arterial (**B**) and coronal reconstruction at portal venous phase (**C**) illustrating the ablation zone.

According to the guidelines of the hospital’s Infection Department prophylactic antibiotic was intravenously administered 45–60 min before MWA session and repeated twice over 24 h. A single operator with 12 years of experience performed all ablation sessions. Microwave ablation was always performed in an inpatient setting under local anesthesia (10cc of 2% Lidocaine Hydrochloric on skin and subcutaneous tissues) and intravenous analgesia (1 gr paracetamol and 100 mg of tramadol diluted in 100 mL of normal saline were administered during the procedure) [14]. Under local sterility, microwave ablation was performed with percutaneous approach in all lesions. Post the initial CT scan, skin entry point was selected. All treatments were performed using the same MWA equipment (16G microwave antenna, HS AMICA, HS HOSPITAL SERVICE SpA, Rome, Italy). The microwave antenna was inserted in the lesion of interest through sequential CT scans. In all lesions included in the present study ablation was performed using a single microwave antenna (Figure 2 and Figure 3). Once in the correct location, the ablation session was set up and performed according to the coagulation charts provided by the manufacturer in consideration of the tumor size and location and the desired safety margin. Whenever deemed necessary, the microwave antenna was re-positioned and a second ablation session was performed, so as to ensure that the final ablation completely encompassed both the target tumor and an annular safety zone around it minimum 5 mm thick. CT scan evaluated any potential immediate complications at the end of the MWA treatment. All patients were hospitalized overnight.

### 2.3. Outcome Measures

**Figure 4:**
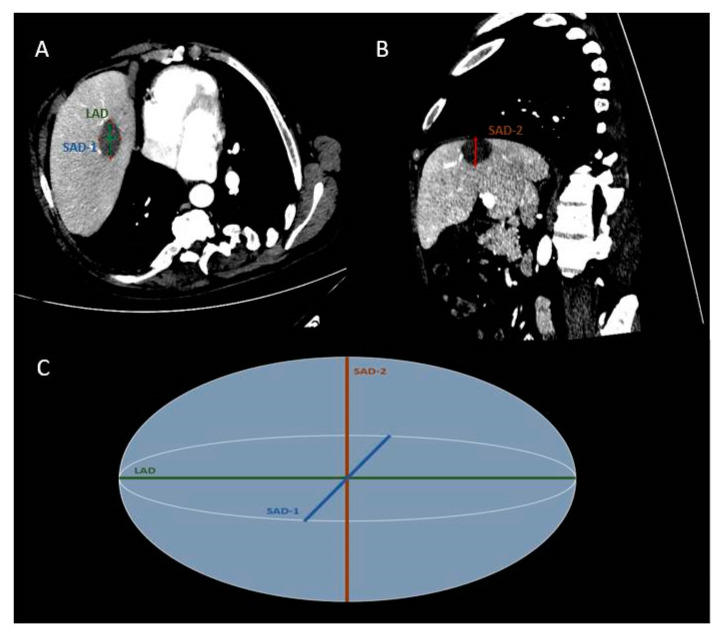
CT scan of a 54-year-old man showing a colorectal cancer metastasis in liver segment VIII post microwave ablation (MWA). Enhanced transverse (**A**) and parasagittal (**B**) CT images. Simplified scheme of MWA zone (**C**). The short-axis diameters (SAD-1,-2) are orientated perpendicular to the long-axis diameter (LAD).

CT (contrast enhanced-in arterial and portal venous phase of enhancement) assessed both the ablation zone size and the potential immediate complications at the end of the ablation treatment. MRI was used at 1, 3 and 6 months for follow-up. The ablated zones were measured lengthwise the antenna pathway, and the diameter of this level of the zone was determined as the long-axis diameter (LAD). The short-axis diameters (SAD-1, SAD-2) were orientated vertical to the long-axis diameter and were measured in two perpendicular axons. (Figure 4). The mean sphericity (mSPH) of the ablation zone was calculated by the ratio among mean short-axis diameter [mSAD = (SAD-1 + SAD-2)/2] and long-axis diameter (LAD), as follows: mSPH = (SAD-1 + SAD-2) / 2LAD. A ratio 1 marks a sphere [10].

### 2.4. Statistical Analysis

Continuous variables were expressed as mean ± standard deviations while categorical variables were expressed as absolute values. Comparisons between HLG and CLG on age and the number of MWA applications were conducted using t-test for independent samples. Comparisons between HLG and CLG on gender and MWA protocol were conducted using χ2 test. To evaluate differences from baseline to follow-up on LAD, SAD-1, SAD-2, mSAD and mSPH, a 2 × 2 (group by time) mixed model analysis of variance (mixed model ANOVA) was conducted (with group as the between-group factor and time as the within-group factor), including gender as covariate (due to significant differences on gender distribution between HLG and CLG). Significant main effects for group and time were followed by independent and dependent t-tests, respectively. Statistically significant group by time interactions were further explored using pairwise comparisons. The statistical threshold was set at *p* < 0.05, with Bonferroni correction for multiple comparisons. All analyses were conducted with IBM SPSS v. 20.

## 3. Results

Demographic and clinical data of patients and lesions included in the present study are presented in Table 1. Neoplasmatic substrate in HLG included: colorectal carcinoma [29/42 (69.05%)], breast adenocarcinoma [5/42 (11.9%)], lung adenocarcinoma [5/42 (11.9%)], pancreatic adenocarcinoma [1/42 (2.38%)], gastric adenocarcinoma [1/42 (2.38%)] and mass-forming cholangiocarcinoma [1/42 (2.38%)]. Details regarding the specific MWA protocol applied in each group by means of watt and duration, as well as the number of applications are shown in Table 1. There were no significant differences in MWA protocol (*p* = 0.253) and MWA applications between HLG and CLG (*p* = 0.286).

### Ablation Zone Parameters

**Figure 5:**
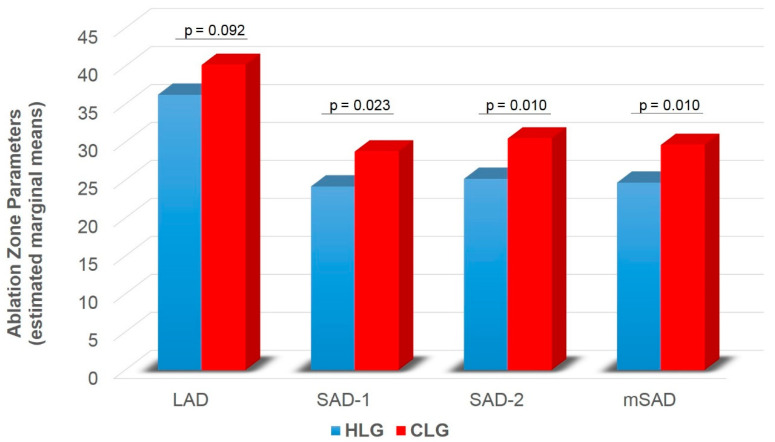
Ablation zone diameter (LAD, SAD-1, SAD-2, mSAD) for HLC and CLG independent of time. LAD = long axis diameter; SAD = short axis diameter; mSAD = mean short axis 1 and short axis 2 diameters; HLC = healthy liver group; CLG = cirrhotic liver group. Values are estimated marginal means following 2 × 2 mixed model ANOVA (group by time), controlling for gender (gender = 1.30). Bonferroni corrected *p*-values are presented for each comparison between HLG and CLG.

**Figure 6:**
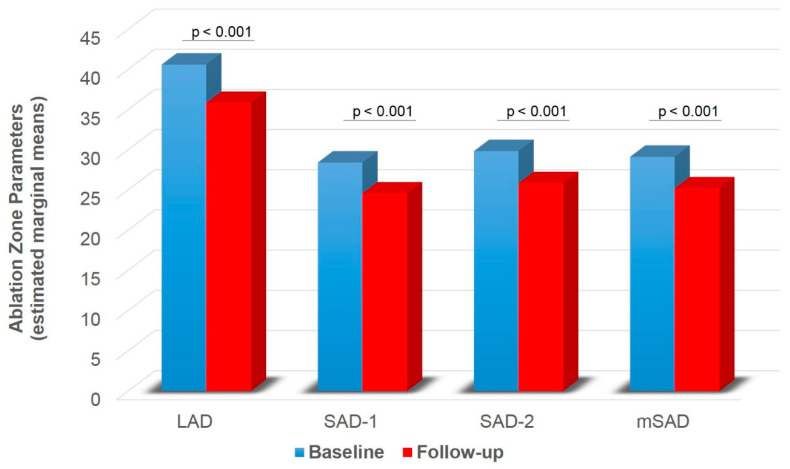
Ablation zone diameter (LAD, SAD-1, SAD-2, mSAD) for baseline and follow-up independent of group. LAD = long axis diameter; SAD = short axis diameter; mSAD = mean short axis 1 and short axis 2 diameters. Values are estimated marginal means following 2 × 2 mixed model ANOVA (group by time), controlling for gender (gender = 1.30). Bonferroni corrected *p*-values are presented for each comparison between baseline and follow-up.

**Figure 7:**
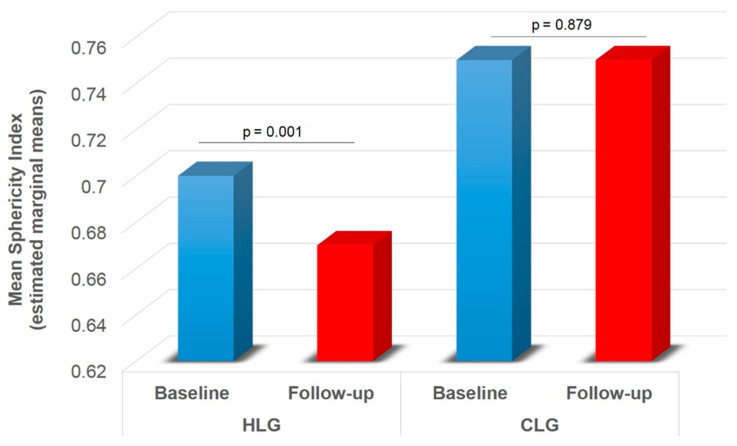
Ablation zone mean sphericity index across HLC and CLG for baseline and follow-up (significant group by time interaction effect). HLC = healthy liver group; CLG = cirrhotic liver group mean short axis 1 and short axis 2 diameters. Values are estimated marginal means following 2 × 2 mixed model ANOVA (group by time), controlling for gender (gender = 1.30). Bonferroni corrected *p*-values are presented for each comparison between baseline and follow-up within each group.

A significant main effect of group was found on SAD-1 (F1,74 = 5.416, *p* = 0.023), SAD-2 (F1,74 = 6.896, *p* = 0.010) and mSAD (F1,74 = 6.943, *p* = 0.010) (Table 2). HLG showed lower SAD-1 (pcorr = 0.023), SAD-2 (pcorr = 0.010) and mSAD (pcorr = 0.010) compared to CLG (Figure 5). A significant main effect of time was also found on LAD (F1,74 = 31.815, *p* < 0.001), SAD-1 (F1,74 = 25.064, *p* < 0.001), SAD-2 (F1,74 = 16.807, *p* < 0.001), mSAD (F1,74 = 27.376, *p* < 0.001) (Table 2). Paired samples t-tests with Bonferroni correction showed decreased values in all indices at follow-up compared to baseline (pcorr < 0.001) (Figure 6). A significant group by time interaction was also found on mSPH (F1,74 = 4.211, *p* = 0.044, Wilk’s lambda = 0.946). Pairwise comparisons were further examined. HLG had significantly lower mSPH at follow-up (pcorr = 0.001) where CLG did not show any significant change (pcorr > 0.05) (Figure 7).

## 4. Discussion

The present study demonstrates that the efficacy of percutaneous MWA of hepatic lesions on terms of sphericity index is comparable when treating tumors developed in cirrhotic or healthy liver parenchyma. Although according to the results of the present study there seems to be a significant difference on the resultant SAD of the ablation zones produced in the cirrhotic liver parenchyma, this has no effect on the sphericity index. The typical MWA zone resulting post a single energy delivery of a single probe is an elongated ellipsoid zone with rotational symmetry around the probe axis, whose aspect ratio (defined as: S = D/L, where L is the maximum ablation size along the probe axis and D is the maximum ablation size perpendicular to it) generally ranges from 0.55 to 0.75 [12]. In the present study sphericity indices of the ablation zones in both healthy (mSPH = 0.70 ± 0.13) and cirrhotic (mSPH = 0.75 mm ± 0.12 mm) liver parenchyma were found in the upper limits of the range proposed by Hoffmann et al. with no significant difference resulting from the between-group comparisons.

Knowing that sphericity index of the expected ablation zone is not related to parenchymal status is essential for the technical and clinical efficacy of percutaneous MWA in both cirrhotic and healthy livers. For hepatocellular carcinoma patients with cirrhotic liver percutaneous ablation serves according to the Barcelona Clinic Liver Cancer (BCLC) classification as an alternative to surgery for very early- and early-stage disease [1,15,16]. On the other hand, in patients with metastatic liver disease where the lesion shape is most commonly spherical it is essential to know that parenchymal status has no effect upon the expected sphericity index [17,18,19].

As it has been shown in the literature, MWA favorably compares with RFA in liver and other locations in the body [1,2,20]. The results of the present study show that the oven effect has neither any relevant technical impact upon the ablation zone sphericity index nor any clinical impact upon the local tumor control. Our results are in agreement with those published by Cassinoto et al. who reported that for radiofrequency ablation there were no differences in the ablation zone volume between cirrhotic and healthy liver parenchyma [11]. Similar to that study, all our measurements concerning the ablation zone sphericity index were performed at the end of the ablation procedure. It has to be noted however, that there is a possible evolution of sphericity with time; as it has been shown in the lung, post MWA ablation zones can vary in the sub-acute setting achieving largest size at seven days post treatment [21].

According to the results of the present study in the one month follow-up there is significant sphericity loss in the ablation zones of healthy liver as opposed to cirrhotic parenchyma where the sphericity index remains stable. One potential explanation for that could involve the resultant contraction during liver ablation which depends not only upon type but also hydration of ablated tissue [22,23,24]. In cirrhotic liver, presence of fibrosis and reduced water content theoretically would contribute towards less contraction as opposed to healthy liver parenchyma.

This study has several limitations including its retrospective design and the number of patients evaluated, which is relatively small, limiting the generalization of our findings. The significant differences in tumor diameters between groups can introduce a bias; additionally, repositioning of the microwave antenna and second ablation can modify the shape of the necrotic zone. Moreover, this paper considers only a small subset of the many available treatment protocols (in terms of power and time settings) and microwave products. Additionally, there was no evaluation of the sphericity index between healthy and cirrhotic groups in the follow-up period. Finally, other ablation techniques including radiofrequency, cryoablation, electochemotherapy and irreversible electroporation have not been evaluated [25].

In conclusion, findings of the present study indicate that although in cirrhotic liver short axis diameters of the MWA zone seem to be significantly longer, this has no effect on the immediate post ablation sphericity index which showed no significant difference between cirrhotic vs. healthy liver lesions. On the contrary, as time passes by, the ablation zone tends to become significantly more ellipsoid in healthy whilst remains stable in cirrhotic liver.

## Figures and Tables

**Table 1:** Demographic and clinical data of the selected patients and lesion after MWA.

Data	Total Group	HLG	CLG	*p*-Value
**Patients (n)**	46	28	18	-
**Lesions (n)**	77	42	35	-
**Age (yrs)**	66.49 ± 9.90	66.90 ± 9.47	66.00 ± 10.50	0.692
**Gender (M/F)**	54/23 (70.1/29.9)	22/20 (52.4/47.6)	32/3 (91.4/8.6)	<0.001
**MWA protocol (A/B/C/D/E/F/G)**	22/23/1/3/25/1/2 (28.57/29.87/1.30/3.90/32.47/1.30/2.60)	15/9/1/1/13/1/2 (35.71/21.43/2.38/2.38/30.95/2.38/4.76)	7/14/0/2/12/0/0 (20.00/40.00/0.00/5.71/34.29/0.00/0.00)	0.253
**MWA applications**	1.36 ± 0.48	1.31 ± 0.48	1.43 ± 0.50	0.286
**Tumor diameter (mm)**	24.18 ± 9.99	20.67 ± 8.84	28.40 ± 9.75	<0.001

Note. MWA = microwave ablation; HLG = healthy liver group; CLG = cirrhotic liver group; n = number; yrs = years; M/F = male/female. Age is expressed as mean ± standard deviation. Qualitative variables (gender, MWA protocol) are presented as proportions (percentages). Patients, lesions and gender are expressed as absolute frequencies. MWA protocol categories include: A = 40 watt/5 min; B = 40 watt/10 min; C = 60 watt/3 min; D = 60 watt/5 min; E = 60 watt/10 min; F = 100 watt/5 min; G = 100 watt/10 min.

**Table 2:** Ablation zone diameter (in mm) and sphericity index for HLG and CLG.

Ablation Zone Parameters	HLG (n = 42)		CLG (n = 35)		Main Effects and Interactions		
	Baseline	Follow-Up	Baseline	Follow-Up	Group	Time	Group by Time
**LAD**	38.76 ± 10.24 (19–60)	34.31 ± 9.80 (15–55)	42.37 ± 8.08 (28–66)	37.29 ± 7.70 (23–62)	F = 2.905, *p* = 0.092	F = 31.815, *p* < 0.001	F = 2.779, *p* = 0.100
**SAD-1**	26.67 ± 8.41 (11–46)	22.69 ± 7.80 (7–40)	30.03 ± 7.75 (14–45)	26.29 ± 7.58 (12–42)	F = 5.416, *p* = 0.023	F = 25.067, *p* < 0.001	F = 0.029, *p* = 0.864
**SAD-2**	27.69 ± 8.78 (12–45)	23.76 ± 8.32 (11–40)	31.86 ± 8.02 (17–48)	27.80 ± 7.07 (14–45)	F = 6.896, *p* = 0.010	F = 16.807, *p* < 0.001	F = 0.182, *p* = 0.671
**mSAD**	27.18 ± 8.26 (13.50–45.50)	23.23 ± 7.73 (11.00–39.50)	30.94 ± 7.14 (17–46.50)	27.04 ± 6.74 (15–43.50)	F = 6.943, *p* = 0.010	F = 27.376, *p* < 0.001	F = 0.038, *p* = 0.846
**mSPH**	0.71 ± 0.13 (0.39–1.00)	0.68 ± 0.13 (0.36–0.94)	0.74 ± 0.13 (0.46–1.00)	0.73 ± 0.15 (0.43–0.98)	F = 3.311, *p* = 0.073	F = 1.214, *p* = 0.274	F = 4.211, *p* = 0.044

Note. HLG = healthy liver group; CLG = cirrhotic liver group; mm = millimeters; LAD = long axis diameter; SAD = short axis diameter; mSAD = mean SAD-1 and SAD-2; mSPH = mean sphericity. Ablation zone parameters for the CLG and HLG are presented as mean ± standard deviation [min-max]. Main effects and interactions were tested in a 2 × 2 mixed model ANOVA (group by time), controlling for gender. Significant main effects and interactions (*p* < 0.05) are presented in bold.

## Data Availability

The data presented in this study are available on request from the corresponding author. The data are not publicly available at the moment since the present study is the basis for the PhD Thesis of the corresponding author.
